# Partial to Total Generation of 3D Transition-Metal Complexes

**DOI:** 10.1021/acs.jctc.4c00775

**Published:** 2024-09-09

**Authors:** Hongni Jin, Kenneth M. Merz

**Affiliations:** † Department of Chemistry, Michigan State University, East Lansing, Michigan 48824, United States; ‡ Department of Biochemistry and Molecular Biology, Michigan State University, East Lansing, Michigan 48824, United States

## Abstract

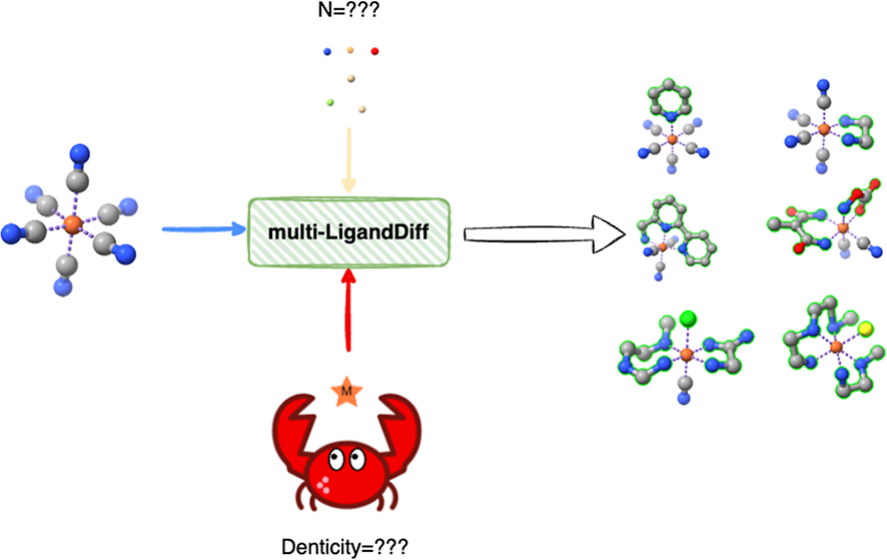

The design of transition-metal complexes (TMCs) has drawn much attention over the years because of their important applications as metallodrugs and functional materials. In this work, we present an extension of our recently reported approach, LigandDiff [Jin et al. *J. Chem. Theory Comput.***20**, 4377(2024)]. The new model, which we call multi-LigandDiff, is more flexible and greatly outperforms its predecessor. This scaffold-based diffusion model allows de novo ligand design with either existing ligands or without any ligand. Moreover, it allows users to predefine the denticity of the generated ligand. Our results indicate that multi-LigandDiff can generate well-defined ligands and is transferable to multiple transition metals and coordination geometries. In terms of its application, multi-LigandDiff successfully designed 338 Fe(II) spin-crossover (SCO) complexes from only 47 experimentally validated SCO complexes. And these generated complexes are configurationally diverse and structurally reasonable. Overall, the results show that multi-LigandDiff is an ideal tool to design novel TMCs from scratch.

## Introduction

Transition-metal complexes (TMCs), sometimes called “Lego molecules”, are a class of compounds composed of small molecules, ions, or atoms arranged around transition metals.^1−3^ Almost any chemical species is capable of coordinating with a transition metal to form TMCs, and TMCs have many unique characteristics due to their complex structures. The bonding patterns of TMCs are different from those of covalent organic compounds. Ligands are bonded to the central metal via dative covalent interactions. And the multiple oxidation states of transition metals give TMCs intriguing redox properties. In addition, the spin state of transition metals varies depending on the electron configuration of the d orbitals, leading to notable magnetic properties. The extensive applications of TMCs in many fields stimulate the ongoing discovery of new TMCs with desirable properties.^4−13^

Over the years, much attention has been focused on the design of novel TMCs. Given this interest, a variety of 3D organometallic generation tools have been developed including conformational- and configurational-based generation algorithms. Typical examples of conformer sampling tools for organometallics are *Architector*(14) and CREST.^15,16^ With a given complex, an ensemble of configurationally identical complexes in different optimized conformations are created. For example, CREST uses metadynamics to explore the conformational space of various chemical systems at the extended tight-binding (xTB) level. This automated and efficient sampling method performs well for structure generation and physicochemical property prediction for drug-like organic molecules.^17^ However, undesirable bond breaking during simulation can be observed for TMC sampling.^18^*Architector*^14^ highlights conformer sampling for f-block systems, which usually have more coordination sites than the more common d-block complexes by using a distance geometry algorithm.^19,20^ Predefined ligands are optimized with force field methods before conformer generation, and the generated conformer ensembles are sorted at the GFN2-xTB level.^21^ Related work based on distance geometry for conformers/stereoisomers of TMCs have also been described recently, such as molAssembler^22^ and MACE.^23^

Another approach, molSimplify,^24^ aims at generating configurationally new TMCs. Users first need to assign ligands that can be either provided by the users or selected from its database. The tool then assembles these assigned ligands into a specific metal. Since the 3D shapes of ligands are predefined, these specified ligands are rigidly attached to the central metal without any geometry optimization. Postoptimization of the generated complexes is necessary for downstream tasks. Similarly, DENOPTIM^25^ uses genetic algorithms to generate hypothetical complexes. Moreover, this method is based on fragment building, i.e., the users still need to provide ligands to build complexes. For the generation of configurationally new organometallics, the obvious drawback of the existing methods is that the generation largely depends on the input of available ligands. Furthermore, these methods are incapable of generating ligands from scratch. These generation tools focus on the recombination of available ligands. The diversity of the generated complexes is already predetermined by the possible combination of ligands.

To overcome limitations, in our prior work, we introduced LigandDiff,^26^ a generative model for the de novo design of novel TMCs. LigandDiff is a scaffold-based diffusion model, and at a specific coordination site, it can generate numerous diverse ligands while keeping the remaining structure unchanged. The so-called diffusion model is inspired by nonequilibrium thermodynamics.^27^ And it has been widely used in chemistry and biochemistry, mainly for organic molecules^28,29^ and proteins.^30^ In the diffusion model, two separate Markov chains are operated sequentially to model the dynamics of the data. In the diffusion step, the forward chain includes a sequence of noised data points via the addition of random noise to the clean input data within the diffusion steps *T*. A neural network ϕ is then trained with the noised data so that it can accurately predict how much noise is added at a specific step *t*. The generation/denoising step reverses the forward chain, and it starts from pure noise sampled from a Gaussian distribution; the parametrized ϕ then predicts and removes the noise from these disordered points step by step and finally generates a realistic output.

In this work, we introduce multi-LigandDiff, which has two improvements over LigandDiff. First, LigandDiff only diffuses/denoises one ligand at each step *t*, which limits the discovery of TMCs. To further increase the flexibility of this model, multi-LigandDiff is capable of generating multiple ligands from a partial generation where at least one ligand already exists in the generated complex to a total generation where only the transition metal is given and all ligands are generated by the model itself to form new TMCs. This improvement greatly increases the diversity of the TMCs generated and makes this model an ideal tool to generate a series of derivatives to investigate the mutual influence of ligands on the transition metal.^31,32^ Second, multi-LigandDiff can generate ligands with a given denticity. Ligand denticity affects organometallics in multiple ways. First, it can control the stability of coordination compounds.^33−35^ Generally, complexes with polydentate ligands are more stable than those with lower denticities, attributed to the chelate effect. Second, they can affect the reactivity of organometallics. For example, for the oxygen reduction reaction, copper complexes with tetradentate ligands have higher catalytic rates than analogues with lower denticity.^36^ And complexes with different ligand denticities also operate via different reaction mechanisms.^37^ In addition, ligand denticity can also be used to tune the geometry of organometallics.^38,39^ The great importance of ligand denticity led us to allow multi-LigandDiff to predefine the denticity of each generated ligand.

As a universal tool for ligand generation based on the scaffold technique, multi-LigandDiff allows for a wide range of applications in transition-metal chemistry. It is an ideal tool to investigate the structure–activity relationship of organometallic compounds in biological systems. A variety of TMCs are being investigated as anticancer agents,^40^ and ligands can tune the structural and physicochemical properties of these drug candidates. For example, arene is a common moiety in osmium drug candidates and studies indicate that arene improves the antiproliferative activity of metallodrugs but at the cost of lower aqueous solubility.^41,42^ While the rational design of arene substituents with desirable properties still requires much effort, multi-LigandDiff can accelerate the high-throughput screening of possible candidates since the automated design without any human expertise saves time and the numerous, diverse outputs can also avoid missing interesting candidates biased by human intuition. In addition, one feature of multi-LigandDiff is the generation of polydentate ligands with the expected denticity. TMCs with multidentate ligands have unique properties. For instance, transition-metal pincer compounds usually have a tridentate ligand coordinated to the metal and form two rings, giving rise to the observed high stability. They are widely used as catalysts for many reactions, such as (de)hydrogenation and cross-coupling.^43^ By specifying the polydentate ligand generation, multi-LigandDiff has the potential to design novel pincer derivatives with higher catalytic activity. Moreover, multi-LigandDiff can also be used to investigate bonding patterns between ligands and metal. Since any atom with donating electrons can coordinate with the metal, multiple coordination modes may exist for a given complex.^44^ Moreover, the flexible electron configurations of the metal centers further enrich the coordination patterns.

The investigation of metal–ligand and ligand–ligand interactions facilitates the optimal design of novel TMCs. The scaffold-based ligand design in multi-LigandDiff opens up an efficient way to provide a series of analogues for research into bonding interactions in transition-metal chemistry. In this work, we give an example of the application of multi-LigandDiff by designing novel spin-crossover (SCO) complexes. SCO compounds are a class of TMCs that can interconvert between a high-spin (HS) state and a low-spin (LS) state under external stimulus.^45^ The transition between both states is likely to happen because the energy gap between both configurations is usually small, typically within 10 kcal/mol.^46^ SCO can lead to dramatic changes in physical properties, such as color, magnetic moment, conductivity, and dielectric constant, making SCO complexes ideal candidates for molecular electronic and spintronic devices.^47,48^ Therefore, the de novo design of novel SCO complexes with switching properties is highly desirable for a range of applications. In this case study, we used multi-LigandDiff to successfully generate hundreds of novel SCO derivatives from experimentally validated SCO complexes.

## Method

### Data Set

The source data^49,50^ reporting all mononuclear TMCs extracted from the Cambridge Structural Database (CSD), were reused by following the procedures proposed in LigandDiff,^26^ i.e., discarding TMCs with missing hydrogen atoms and disorder. In order to curate more diverse complexes, we extended the upper bound of the system size from 100 in LigandDiff to 200 in this work. Based on the element analysis for TMCs in the CSD, the element set S = {H, C, N, O, F, P, S, Cl, Br, Cr, Mn, Fe, Co, Ni, Cu, Zn} includes most common elements in TMCs, and we then excluded TMCs, which included element beyond S, finally giving rise to a set of 30,118 mononuclear octahedral TMCs as the base set of curated complexes with at least one ligand. The combinations and permutations method was applied to do multiligand masking. For each complex, we started by masking one ligand, then gradually increased to two ligands, and so on until all ligands were masked. The masked ligands are used for diffusion, i.e., random noise is added to each masked ligand. The unmasked ligands and transition metals are unchanged. For a given complex with six monodentate ligands, 63 samples could be obtained, i.e., . With such a design, we finally obtained 404,126 complexes. All hydrogen atoms were removed to reduce the computational cost. Two subsets with 1000 complexes were used for validation and testing, while the remaining data were used for training. To check the transferability of multi-LigandDiff, we used our previous curated PPR_100 set, which includes 100 Pt, Pd, and Ru complexes with more than 50 atoms.^26^ The three transition metals are not included in the training data set, which makes it challenging for multi-LigandDiff to generate realistic ligands under unfamiliar conditions.

### Molecule Representation

Each TMC is denoted as a point cloud *x* = [*r*,*h*_a_,*h*_L_,*h*_c_], where *r* denotes the atom coordinates  and *h*_a_ denotes the one-hot representations of atom type , *N* is the total number of atoms, *m* is the number of atom types. *h*_L_ is one-hot embedding to encode the ligand information, , where *l* is the number of ligands. *h*_c_ is one-hot embedding to encode the coordination site, . *h*_L_ and *h*_c_ are concatenated together as *h*_Lc_ for the sake of simplicity. A scaled ratio of [0.1, 0.25, 1] is applied to *x* = [*r*,*h*_a_,*h*_Lc_] to highlight the importance of *h*_Lc_, which is pivotal in ligand generation, as illustrated below. The noise is only relevant to *r* and *h*_a_, i.e., in the diffusion process, the sampled noise is added to *r* and *h*_a_ from the diffused ligands and in the denoising step, randomly generated noise is used to represent *r* and *h*_a_ for the generated ligands while *h*_Lc_ is already predefined before denoising.

### 3D-Conditional Diffusion Models

The assigned ligand *x*^L^ is diffused/generated under a fixed context *u*, consisting of the unmasked ligands and the central metal. The context *u* has the same embedding components as *x*^L^. Multi-LigandDiff uses the same architecture as LigandDiff to estimate the noise . The neural network ϕ uses geometric vector perceptron (GVP)^51,52^ to update each atom in a given compound. Similar work has indicated that GVP is more expressive than the popular equivariant graph neural network.^53^ GVP defines both scalar features, , and vector features, , for each embedding, and both types of features are updated through separate nonlinear transformations and mutual information exchange between *s* and *V* to extensively extract information from each compound. The interactions between atoms are modeled via message passing and aggregation from neighbors. Any atom except the central atom itself is considered a neighbor to capture the global structure of each compound. We refer readers to LigandDiff^26^ for more details about ϕ.

### Ligand Generation Controlled by LD_g_

To further increase the diversity of generated ligands, multi-LigandDiff first introduces CN_g_ to predefine the coordination number of generated ligands, CN_g_ = 6 – CN_c_, where CN_c_ is the sum of ligand denticities in context *u*. For a given complex with CN_c_ = 3, the corresponding CN_g_ is 3, and it has three different ligand-denticity (LD_g_) combinations for the generated ligands because the model can generate (i) one tridentate ligand (LD_g_ = 3); (ii) one bidentate ligand and one monodentate ligand (LD_g_ = 21); and (iii) three monodentate ligands (LD_g_ = 111). The length of LD_g_ indicates the number of generated ligands, and each number in LD_g_ indicates the denticity of the generated ligand. For total generation, where CN_g_ = 6, i.e., only the metal is in context, all ligands are generated by the model itself, and 11 types of LD_g_ are derived. In total, 18 different LD_g_ can be chosen for partial generation, where CN_g_ < 6. The full list of LD_g_ values is given in Table S1. With this implementation, a reference complex with CN_c_ = 3 can derive 3 different configurations with respect to ligand denticity, and with the same denticity, each generated ligand has numerous variations. Both types of diversity enrich the ensemble of generated TMCs.

Once the LD_g_ is determined, the ligand generation process starts from the random initialization of generated ligands. The model generates an ensemble of random data points to represent [*r*,*h*_a_] for each generated ligand with a specified ligand size. The assigned LD_g_ is then encoded into *h*_c_ to specify the expected denticities of the generated ligands. Meanwhile, *h*_L_ is also encoded for the generated ligands. With the entire embedding *x* = [*r*,*h*_a_,*h*_L_,*h*_c_], abbreviated as *x* = [*r*,*h*_a_,*h*_Lc_], the model then starts to denoise [*r*,*h*_a_] in a stepwise manner via the neural network ϕ to finally generate chemically realistic ligands. The entire process is completely automated without any human intervention, which makes the ligand-generation process efficient.

### Design of Spin-Crossover Complexes

**Figure 1:**
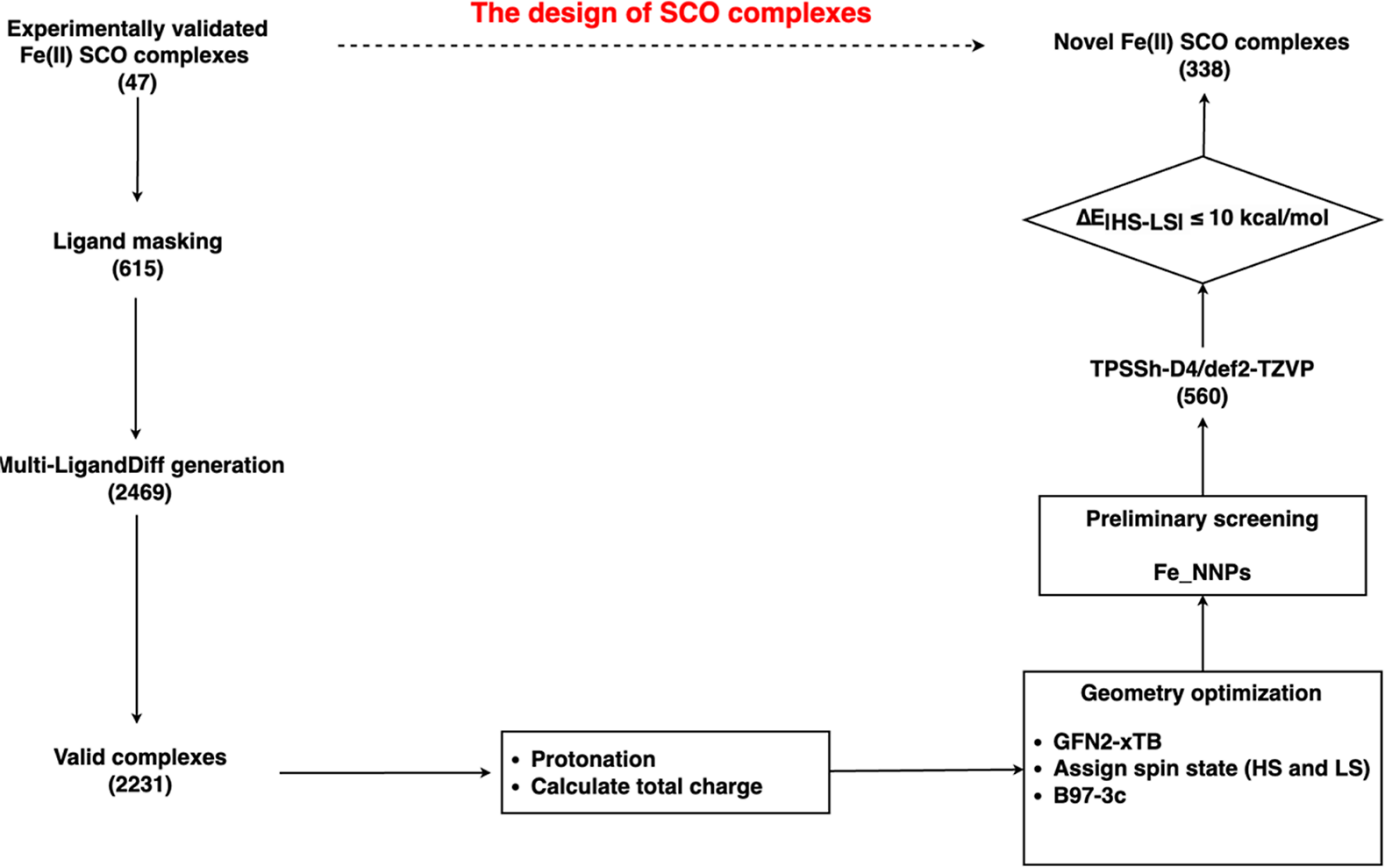
Overview of Fe(II) SCO complexes design.

**Figure 2:**
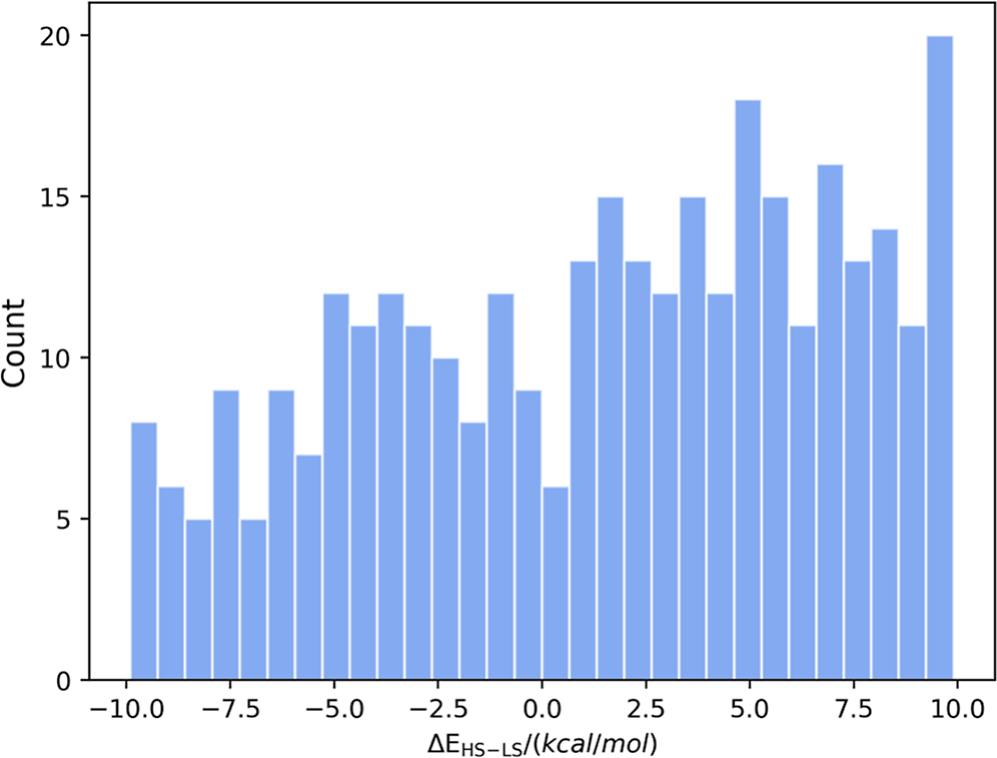
Histogram of spin splitting energies found in the GEN_SCO_338 data set.

Vennelakanti and co-workers recently curated a set of 95 experimentally validated SCO complexes, SCO-95,^54^ from which we randomly extracted 47 Fe(II) SCO complexes (SCO_47) with diverse coordination types (Table S2). 615 samples were obtained by masking ligands from partially to totally in these 47 complexes. For example, given a complex which has only 2 tridentate ligands, we can get 3 samples, of which two samples S1, S2 are from the partial masking of only one ligand with CN_g_ = 3, and the third sample S3 is obtained by masking both tridentate ligands with CN_g_ = 6. Depending on CN_g_, each sample may have multiple variations to generate. For example, either S1 or S2 with CN_g_ = 3 can give three variations with LD_g_ = 111/21/3 and S3 with CN_g_ = 6 can get 11 variations, as described in the section above. Therefore, given a complex with two tridentate ligands, multi-Ligand can obtain 17 variations for the ligand generations. With this strategy, a total of 2469 variations were obtained from these 615 samples. And for each of them, we assigned the size of each generated ligand to be 10 heavy atoms or less. Finally, the model generated 2231 valid complexes. We then added hydrogen atoms back to each valid complex and calculated the total charge of each compound. Next, each complex was first optimized at the GFN2-xTB^21^ semiempirical level using *xtb*(55) version 6.6.1. Then each complex was manually assigned both HS state and LS state and was further optimized at B97-3c level^56^ using ORCA 5.0.4^57^ with the *TightSCF*, *SlowConv*, and *SOSCF* settings. Energy screening was conducted for the configurations that have successfully optimized geometries in both spin states. In our prior work, we developed some neural networks to model the energetics of TMCs.^58,59^ To accelerate the high-throughput screening of SCO complexes, we first used our previously developed Fe_NNPs^58^ model to do preliminary screening. The Fe_NNPs model is a deep learning method that can quickly and accurately predict the ground spin state and energetics of Fe(II) complexes. However, the Fe_NNPs model can cover only some generated complexes due to limitations of the element type and system size. For the remaining complexes and complexes that passed the Fe_NNPs screening (560 complexes in all), we calculated the energy gap between both spin states using TPSSh^60^ with the D4 correction^61^ and the def2-TZVP^62^ basis set via ORCA 5.0.4. The RI-J approximation^63^ was used to accelerate the calculations with the def2/J^64^ auxiliary basis set. Finally, 338 complexes were identified as potential SCO complexes, i.e., the energy gaps of which are no more than 10 kcal/mol. Importantly, due to the random initialization of the denoising process, each run of this workflow will generate a new ensemble of complexes and therefore theoretically multi-LigandDiff can generate numerous SCO complexes. We refer to the 338 generated complexes as GEN_SCO_338 and SCO-95 as EXP_SCO_95 to differentiate the original source of both sets in this work. The complete workflow is given in Figure 1. The spin splitting energies of GEN_SCO_338 are given in Figure 2. In the GEN_SCO_338 set, 134 complexes are in the HS ground state while 204 complexes are in LS ground state.

## Results and Discussion

### General Sampling

#### Multi-LigandDiff vs LigandDiff

**Figure 3:**
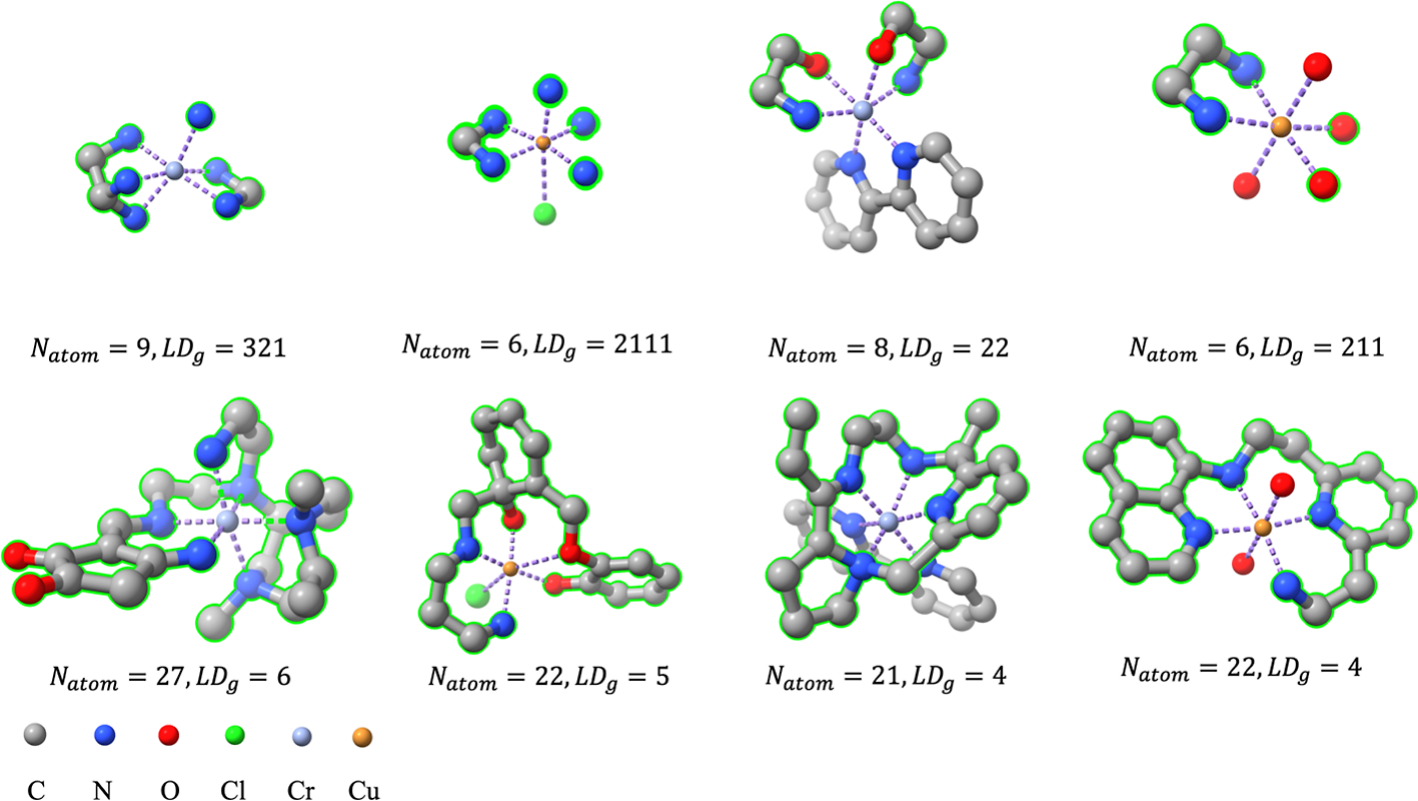
Complexes generated with the same predefined ligand denticity but different assigned ligand size. The actual ligand denticity and the assigned ligand size are labeled as LD_g_ and *N*_atom_, respectively. Complexes in each column were generated in the same context. Complexes (top) have unexpected ligand denticity due to the small, assigned ligand size while complexes (bottom) have polydentate ligand with expected denticity by specifying a large ligand size. The generated ligands are highlighted in green outline.

We first compared multi-LigandDiff with LigandDiff. The test set split in this work was used to evaluate the performance of multi-LigandDiff and LigandDiff. Since LigandDiff can only generate one ligand, for each complex in the test set we generated one ligand with maximum denticity using multi-LigandDiff. For example, for a given sample with CN_g_ = 3, multi-LigandDiff only generated a tridentate ligand with the expected LD_g_ = 3. Each generated ligand was randomly assigned a size in the range 6–20, as we did in our previous LigandDiff work. We compared the performances of both models by evaluating the metrics used in LigandDiff,^26^ including (1) the validity of generated ligands *p*_l_^val^, i.e., the ratio of chemically valid ligands in all generated ligands; (2) the connectivity of generated ligands *p*_l_^con^, i.e., the ratio of connected ligands in all valid ligands; (3) the validity of the whole complex *p*_c_^val^, i.e., the ratio of valid complexes in all generated complexes; (4) the novelty of generated ligands *p*_l_^nov^, i.e., the ratio of generated ligands that do not appear in the training set to all generated ligands; and (5) the uniqueness of the generated ligands *p*_l_^uniq^, i.e., the ratio of unique ligands to all generated ligands. (1) and (2) are checked by RDKit, same for (4) and (5), for which we compare SMILES of the generated ligands, while (3) is obtained using molSimplify. We noticed that in the successfully generated valid complexes from multi-LigandDiff, some polydentate ligands (LD ≥ 4) have unexpected ligand denticity due to the mismatch of the assigned ligand size with regard to the ligand denticity. The assigned ligand is too small to meet the expected denticity. For such cases, multi-LigandDiff generated several ligands with a lower denticity to replace a polydentate ligand. To validate this hypothesis, the relationship between the ligand size and ligand denticity in the training data set was analyzed. It is clear that polydentate ligands usually have more than 20 heavy atoms (Figure S1). Moreover, this hypothesis can be further validated by increasing the ligand size of the polydentate ligand. Given a complex with a predefined polydentate ligand to generate, unexpected ligand denticity can be avoided by assigning a large ligand size (Figure 3). Next, to evaluate the overall performance of multi-LigandDiff, we then generated all possible samples for each CN_g_ in the test set, leading to 4057 samples. To match the size of ligands with regard to the ligand denticity in the diffused samples (Figure S1), for each generated ligand in each sample the sampled ligand size is less than 10 heavy atoms if the generated ligand is expected to be monodentate or bidentate; otherwise, the assigned ligand size is in the range of 10–30. Finally, to check the transferability of multi-LigandDiff, a total of 5612 samples were generated by enumerating all possible LD_g_ values for each given CN_g_ in the ppr_100 set. All results are listed in Table 1. Multi-LigandDiff clearly outperforms LigandDiff. LigandDiff mainly focuses on monodentate ligand generation. However, for the 1000 samples in the test set, only 218 samples have just one monodentate ligand to generate, and each sample also has a large context size, which is beyond the domain of LigandDiff. Both factors lead to the poor performance of LigandDiff. In contrast, multi-LigandDiff generates nearly 99% valid and connected ligands in all three different cases. And the generated ligands from nearly 89% of the samples are well incorporated into the given context to form chemically valid complexes. The generated ligands in each valid complex are configurationally different from the diffused ligands in the training data set, indicated by *p*_l_^nov^. In addition, these generated ligands are highly unique in each valid complex, which indicates that multi-LigandDiff is able to differentiate the given context in each sample and generate the corresponding appropriate ligands. The remarkable performance of PPR_100 indicates that multi-LigandDiff has good transferability and can be used to generate reasonable complexes for any transition metal.

**Table 1:** Validity, Connectivity, Novelty, and Uniqueness of Generated Ligands and Validity of Generated Complexes^a^

model	*N*_sample_	*N*_atoms_	*p*_l_^val^	*p*_l_^con^	*p*_c_^val^	*p*_l_^nov^	*p*_l_^uniq^
LigandDiff	1000	6–20	0.97 ± 0.006	0.75 ± 0.006	0.72 ± 0.009	0.95 ± 0.007	0.96 ± 0.007
multi-LigandDiff (max LD_g_)	1000	6–20	0.99 ± 0.002	0.99 ± 0.002	0.93 ± 0.005	1.00 ± 0	0.96 ± 0.006
multi-LigandDiff (all LD_g_)	4057	LD ∼ 10 (LD < 3)	0.99 ± 0.002	0.98 ± 0.002	0.87 ± 0.004	1.00 ± 0	0.96 ± 0.003
		10–30 (LD ≥ 3)					
LigandDiff (PPR_100)^b^	148	11–40	0.94 ± 0.017	0.94 ± 0.015	0.87 ± 0.026	1.0 ± 0	0.92 ± 0.019
multi-LigandDiff (PPR_100)	5612	LD ∼ 10 (LD < 3)	0.99 ± 0.001	0.99 ± 0.001	0.88 ± 0.005	1.00 ± 0	0.96 ± 0.002
		10–30 (LD ≥ 3)					
multi-LigandDiff (CN = 3, 4, 5)	4609	LD ∼ 10 (LD < 3)	0.99 ± 0.001	0.99 ± 0.001	0.83 ± 0.003	1.00 ± 0	0.84 ± 0.003
		10–30 (LD ≥ 3)					

a The results are reported as “mean ± std” over 10 independent runs. LD is the assigned ligand denticity of each generated ligand.

b Ref (26).

#### Design of Nonoctahedral Complexes

**Figure 4:**
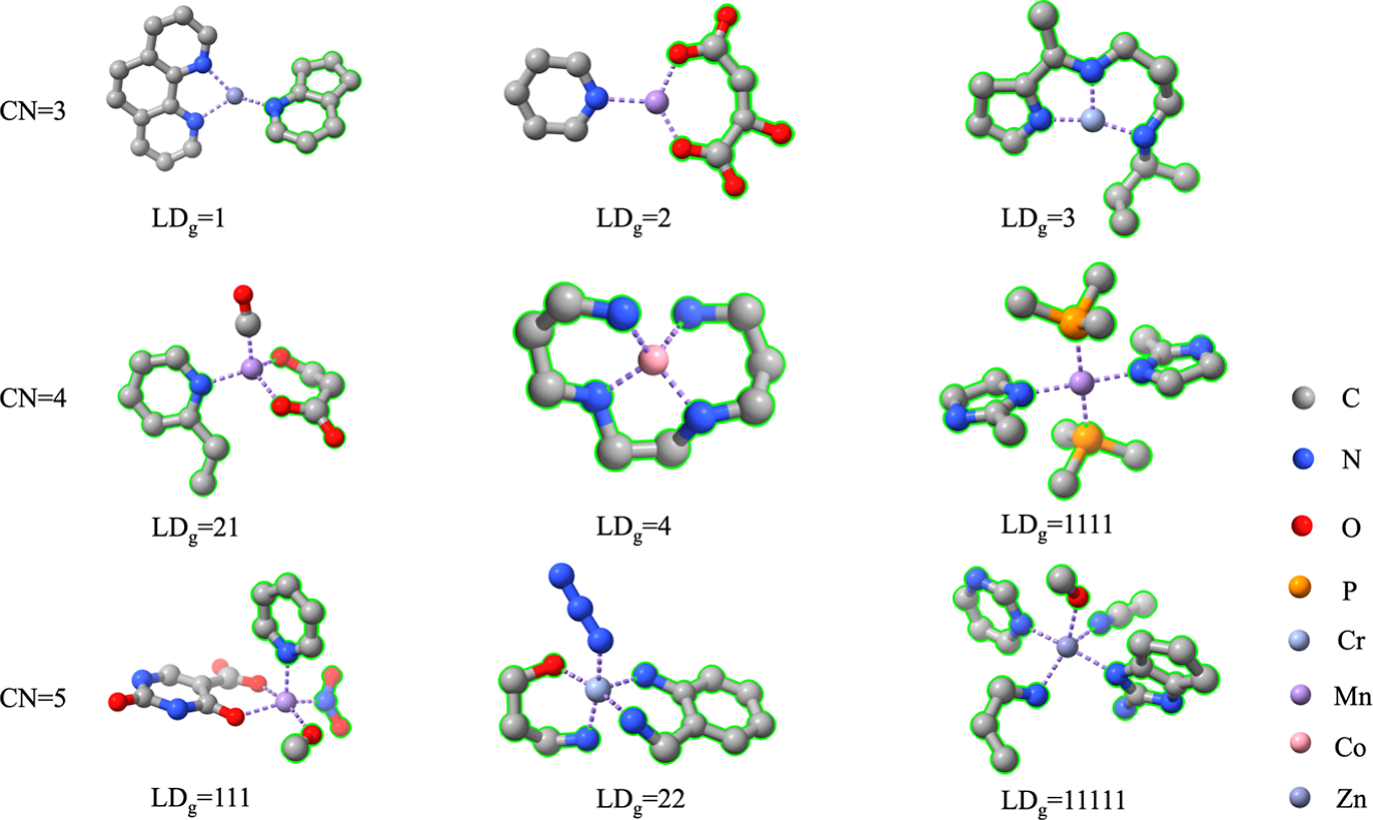
Generated complexes with nonoctahedral geometries.

Although multi-LigandDiff was trained with mononuclear octahedral complexes (CN = 6), to further evaluate the transferability of multi-LigandDiff we tested the ability of the model to generate complexes with CN = 3, 4, 5. In the test set, 868 samples have 0 ≤ CN_c_ < 5. Again, for each sample, ligands with all possible LD_g_ (Table S3) were generated, leading to a total of 4609 samples. The results are given in Table 1. Multi-LigandDiff is also capable of generating realistic complexes with nonoctahedral geometries. In addition, the generated complexes adopt standard structures (Figure 4), e.g., CN = 3 (trigonal planar), CN = 4 (tetrahedral and square planar), and CN = 5 (trigonal bipyramidal and square pyramidal), which indicates that multi-LigandDiff learns the metal–ligand bonding mechanisms in transition metal chemistry and generates chemically reasonable and well-distributed ligands to stabilize the entire structure.

### Design of SCO Complexes

#### Diversity of Coordination Geometries in GEN_SCO_338

**Figure 5:**
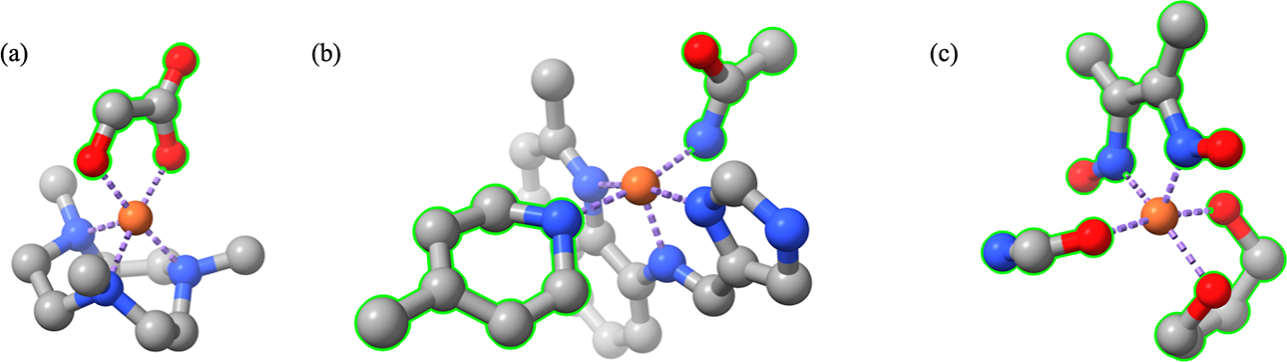
Generated structures with CN = 5. (a) Expected LD_g_ = 3, but since the assigned size of this tridentate ligand is only 5; the model generated a bidentate ligand with the specified size. (b) Expected LD_g_ = 21, the expected bidentate ligand is assigned with only 4 atoms; the model generated a monodentate ligand. (c) Expected LD_g_ = 222, this is a total generation and one of the expected bidentate ligands has only 3 atoms; the model generated a monodentate ligand.

The ligand denticity of GEN_SCO_338 is given in Table 2. Thirteen complexes have new coordination geometries with CN = 5. The ligand size is randomly specified from 1 to 10, and it is not restricted with respect to ligand denticity. But polydentate ligands usually have more atoms than monodentate ligands to stabilize the structure. A possible outcome of this random generation is that the randomly generated ligand is too small to meet the requirement of a polydentate ligand. Figure 5 shows 3 SCO complexes with CN = 5, and each of them has a generated ligand with unexpected denticity due to the small, predefined size of this generated ligand. In terms of CN = 6, GEN_SCO_338 also includes some new coordination types that are not included in SCO_47, such as (1,1,1,1,2) and (1,2,3), which further increases the diversity of coordination geometries in SCO complexes.

**Table 2:** Ligand Denticity of GEN_SCO_338 Set

CN = 5		CN = 6	
ligand denticity	count	ligand denticity	count
1,1,1,1,1	7	1,1,1,1,1,1	47
1,1,1,2	3	1,1,1,1,2	76
1,2,2	1	1,1,2,2	70
1,1,3	1	1,1,1,3	45
2,3	1	1,1,4	8
		1,2,3	38
		2,2,2	29
		2,4	3
		3,3	9

#### Configuration Diversity of GEN_SCO_338

**Figure 6:**
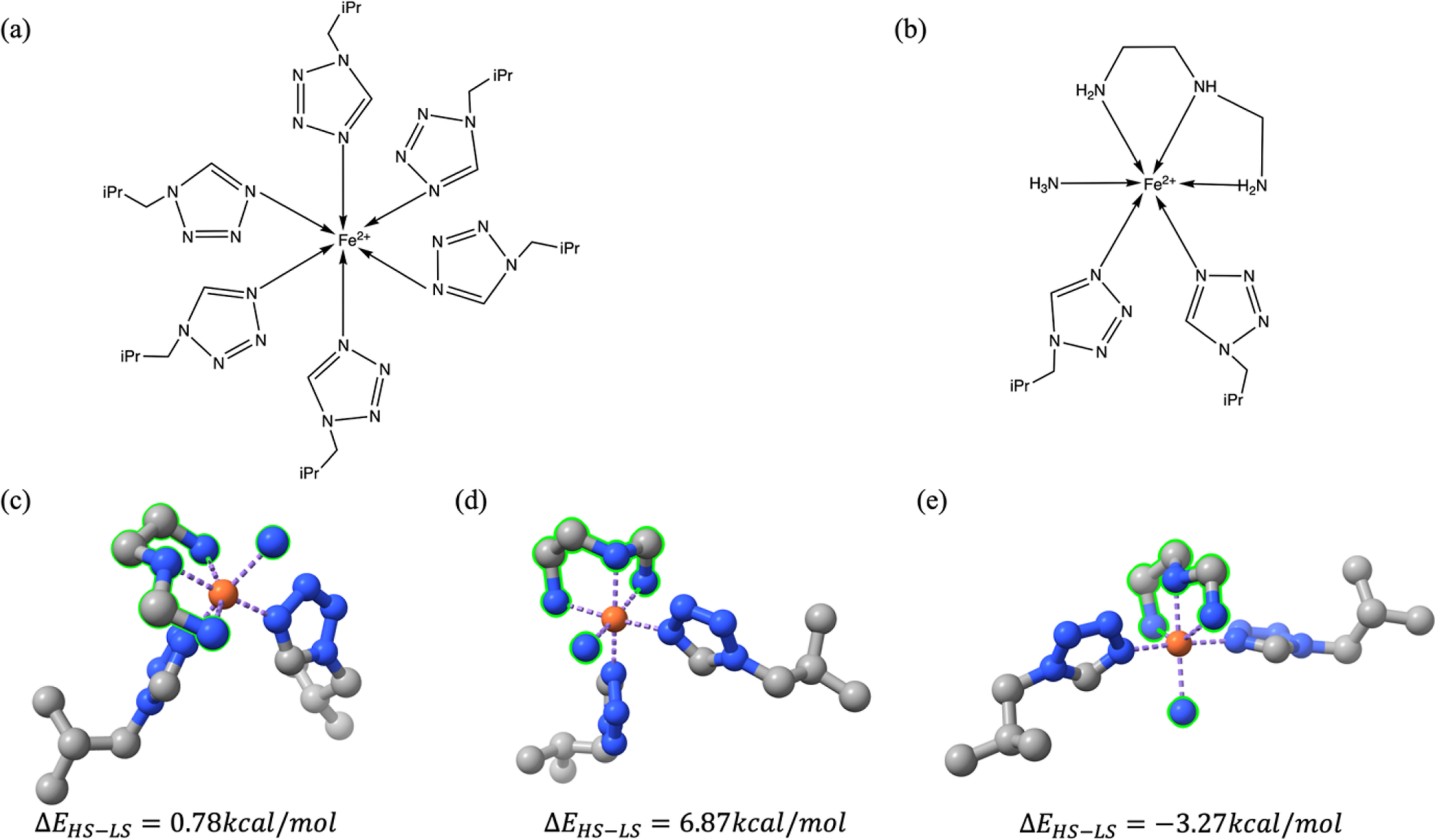
SCO conformers. (a) 2D reference structure (refcode: NIGXUY) (b) 2D generated derivatives of reference structure. (c–e) 3D structures of derivatives in LS state, RMSD: (c) vs (d) = 3.18 Å; (c) vs (e) = 4.52 Å; (d) vs (e) = 2.95 Å.

To evaluate the diversity of configurations in GEN_SCO_338, we checked the uniqueness of ligand configurations in SMILES. Only two reference structures (refcode: NIGXUY and YAGYIP) in SCO_47 have several configurationally identical but conformationally different derivatives in GEN_SCO_338. The NIGXUY complex has three distinctively different conformations, along with different energy splitting gaps (Figure 6), and the YAGIP complex has two distinctive conformers (Figure S2). The three derivatives of the NIGXUY complex have different ground spin states, which indicates the effects of conformational change on the stability of the coordination geometry.^58^ The remaining 333 complexes are unique, and each of them has configurationally different ligands.

#### Bond Lengths in GEN_SCO_338

**Figure 7:**
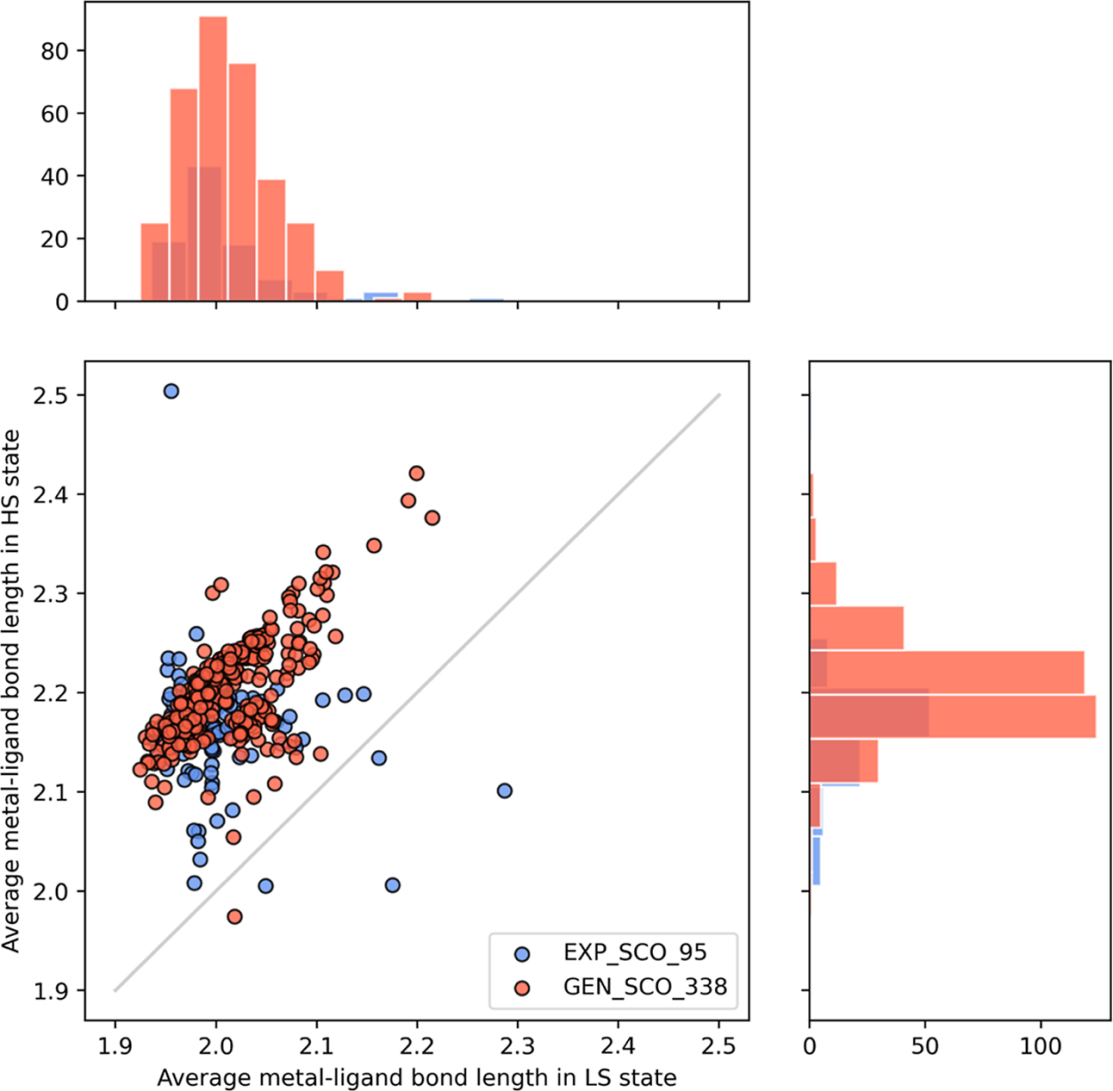
Distribution of metal–ligand bond length in GEN_SCO_338 vs EXP_SCO_95 in both HS state and LS state.

We compared the metal–ligand bond length of GEN_SCO_338 with that of EXP_SCO_95 (Figure 7). The distribution of bond lengths of the generated complexes matches the range of bond lengths of the experimentally synthesized SCO complexes. The bond lengths in both sets range from 1.92 to 2.50 Å. Most complexes in both sets have an average bond length of 1.96–2.06 Å in the LS state and 2.18–2.24 Å in the HS state. GEN_SCO_338 also follows the trend that bond length in the HS state is usually longer than that in the LS state (an increase of ∼0.2 Å from LS to HS), which indicates the spin transition between both states. The only outlier is a five-coordinated complex (Figure 5c), with an average bond length of 1.97 Å in the HS state and 2.01 Å in the LS state. The similar trends in both sets indicate that complexes in GEN_SCO_338 are chemically realistic and experimentally reasonable.

#### Partial Generation vs Total Generation in GEN_SCO_338

**Figure 8:**
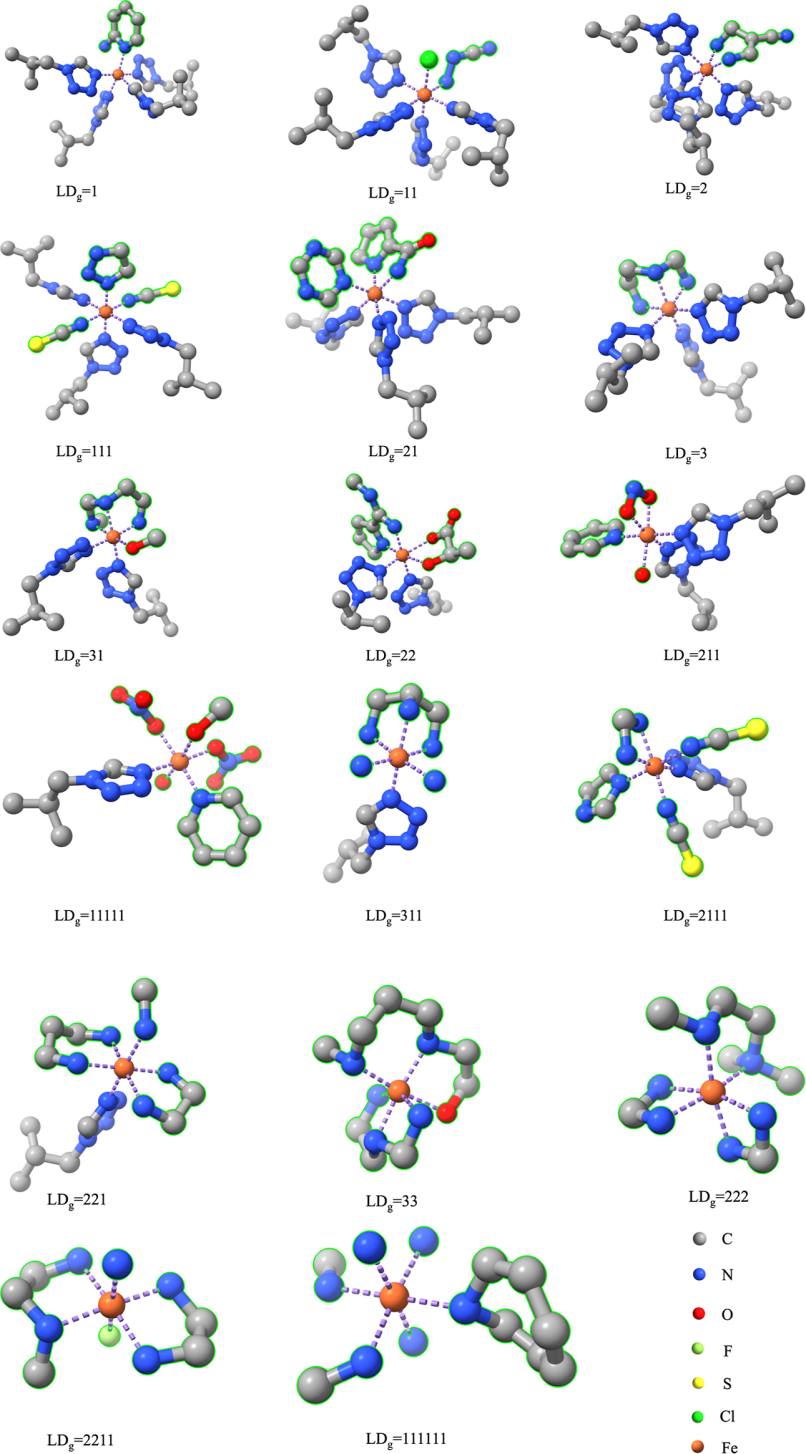
Multiple derivatives of NIGXUY reference structure with different denticities of generated ligands from partial generation to total generation.

338 complexes were generated from 43 reference structures in SCO_47, among which 71 complexes were designed by total generation, i.e., all ligands in these complexes were designed by the model itself. The ligand denticities of the 71 totally generated complexes (Table S4) cover all types with CN = 6, given in Table 2, which indicates that the model is highly capable of designing diverse TMCs without the introduction of any existing ligands. Each of 5 reference structures (refcode: NIGXUY, GOGSAZ, YAGYIP, PEJQIF, and LAGJEK) has more than 10 derivatives in GEN_SCO_338 predominantly because each of them has six configurationally identical, monodentate ligands, giving rise to 63 different types of masking, and each masking also has at least one coordination type for the generated ligands, which greatly increases the possibility and diversity of realistic TMC derivatives. Figure 8 shows some selected derivatives of the NIGXUY reference structure (Figure 6a). Each of them has realistic ligands with the expected denticity. However, polydentate ligands are not included in these complexes because the assigned ligand size is smaller than the expected ligand size for a polydentate ligand, which is usually more than 15 heavy atoms (Figure S1).

## Conclusions

TMCs are potential candidates for metallodrugs, catalysts, and functional materials. However, the design of TMCs with desirable properties is challenging due to the complex bonding patterns and the interplay between oxidation, spin states, and electron configurations. To accelerate the discovery in transition metal chemistry, multi-LigandDiff provides an efficient way to de novo design realistic ligands to form new complexes. Multi-LigandDiff is an extension of LigandDiff^26^ with significant improvements. The model can generate new ligands for incomplete complexes with existing ligands or just the transition metal and output complete complexes. It also allows users to predefine the ligand denticity of the generated ligands and gives realistic outputs. The results indicate that multi-LigandDiff is capable of generating realistic and unique ligands under a given context. And the proposed workflow of Fe(II) SCO complex design validates the potential of multi-LigandDiff for practical applications.

## Data Availability

All data and code are available at https://github.com/Neon8988/multi_LigandDiff

## Supporting Information Available

Size distributions of the diffused ligands in training data set; full list of LD_g_; coordination type of 47 experimentally validated SCO complexes; generated isomers of YAGYIP complex; and ligand denticities of 71 SCO complexes from total generation (PDF)
338 generated SCO complexes (ZIP)

## References

[ref1] Frenking G.; Fröhlich N. The Nature of the Bonding in Transition-Metal Compounds. Chem. Rev. 2000, 100, 717–774. 10.1021/cr980401l. 11749249

[ref2] Reinholdt A.; Bendix J. Transition Metal Carbide Complexes. Chem. Rev. 2022, 122, 830–902. 10.1021/acs.chemrev.1c00404. 34797626

[ref3] Frenking G.; Fernández I.; Holzmann N.; Pan S.; Krossing I.; Zhou M. Metal–CO Bonding in Mononuclear Transition Metal Carbonyl Complexes. JACS Au 2021, 1, 623–645. 10.1021/jacsau.1c00106. 34467324 PMC8395605

[ref4] Malinowski J.; Zych D.; Jacewicz D.; Gawdzik B.; Drzeżdżon J. Application of Coordination Compounds with Transition Metal Ions in the Chemical Industry—a Review. Int. J. Mol. Sci. 2020, 21, 5443 10.3390/ijms21155443. 32751682 PMC7432526

[ref5] Haas K. L.; Franz K. J. Application of Metal Coordination Chemistry to Explore and Manipulate Cell Biology. Chem. Rev. 2009, 109 (10), 4921–4960. 10.1021/cr900134a. 19715312 PMC2761982

[ref6] Renfrew A. K. Transition Metal Complexes with Bioactive Ligands: Mechanisms for Selective Ligand Release and Applications for Drug Delivery. Metallomics 2014, 6, 1324–1335. 10.1039/C4MT00069B. 24850462

[ref7] Lee L. C.-C.; Lo K. K.-W. Luminescent and Photofunctional Transition Metal Complexes: From Molecular Design to Diagnostic and Therapeutic Applications. J. Am. Chem. Soc. 2022, 144, 14420–14440. 10.1021/jacs.2c03437. 35925792

[ref8] Xiao M.; Wu Q.; Li L.; Mu S.; Sørensen M. N.; Wang W.; Cui C. Regenerable Catalyst for Highly Alkaline Water Oxidation. ACS Energy Lett. 2021, 6, 1677–1683. 10.1021/acsenergylett.1c00127.

[ref9] Liu Y.; Bai Y. Design and Engineering of Metal Catalysts for Bio-Orthogonal Catalysis in Living Systems. ACS Appl. Bio Mater. 2020, 3, 4717–4746. 10.1021/acsabm.0c00581. 35021720

[ref10] Raj P.; Singh A.; Singh A.; Singh N. Syntheses and Photophysical Properties of Schiff Base Ni (II) Complexes: Application for Sustainable Antibacterial Activity and Cytotoxicity. ACS Sustainable Chem. Eng. 2017, 5, 6070–6080. 10.1021/acssuschemeng.7b00963.

[ref11] Ugwu D. I.; Conradie J. Anticancer Properties of Complexes Derived from Bidentate Ligands. J. Inorg. Biochem. 2023, 246, 112268 10.1016/j.jinorgbio.2023.112268. 37301166

[ref12] Tisovský P.; Donovalová J.; Kožíšek J.; Horváth M.; Gáplovský A. Reversible on/off and off/on, Light-Stimulated Binding, or Release Processes of Metal Cations from Isatin Diarylhydrazone Complexes in Solution. J. Photochem. Photobiol., A 2022, 427, 113827 10.1016/j.jphotochem.2022.113827.

[ref13] Malviya R.; Singh A. K.; Yadav D. Advances in Metallodrug-driven Combination Therapy for Treatment of Cancer. In Multi-Drug Resistance in Cancer; Wiley, 2023; pp 155–170. 10.1002/9781394209866.ch8.

[ref14] Taylor M. G.; Burrill D. J.; Janssen J.; Batista E. R.; Perez D.; Yang P. Architector for High-Throughput Cross-Periodic Table 3D Complex Building. Nat. Commun. 2023, 14, 2786 10.1038/s41467-023-38169-2. 37188661 PMC10185541

[ref15] Pracht P.; Bohle F.; Grimme S. Automated exploration of the low-energy chemical space with fast quantum chemical methods. Phys. Chem. Chem. Phys. 2020, 22, 7169–7192. 10.1039/C9CP06869D. 32073075

[ref16] Pracht P.; Grimme S.; Bannwarth C.; Bohle F.; Ehlert S.; Feldmann G.; Gorges J.; Müller M.; Neudecker T.; Plett C.; Spicher S.; Steinbach P.; Wesołowski P. A.; Zeller F. CREST—A program for the exploration of low-energy molecular chemical space. J. Chem. Phys. 2024, 160, 114100 10.1063/5.0197592. 38511658

[ref17] Das S.; Merz K. M. Molecular Gas-Phase Conformational Ensembles. J. Chem. Inf. Model. 2024, 64, 749–760. 10.1021/acs.jcim.3c01309. 38206321

[ref18] Bursch M.; Hansen A.; Pracht P.; Kohn J. T.; Grimme S. Theoretical study on conformational energies of transition metal complexes. Phys. Chem. Chem. Phys. 2021, 23, 287–299. 10.1039/D0CP04696E. 33336657

[ref19] Blaney J. M.; Dixon J. S. Distance geometry in molecular modeling. In Reviews in Computational Chemistry; Wiley, 1994; Vol. 5; pp 299–335. 10.1002/9780470125823.ch6.

[ref20] Crippen G. M.; Havel T. F. Distance Geometry and Molecular Conformation; John Wiley & Sons, 1988.

[ref21] Bannwarth C.; Ehlert S.; Grimme S. GFN2-xTB—an accurate and broadly parametrized self-consistent tight-binding quantum chemical method with multipole electrostatics and density-dependent dispersion contributions. J. Chem. Theory Comput. 2019, 15, 1652–1671. 10.1021/acs.jctc.8b01176. 30741547

[ref22] Sobez J.-G.; Reiher M. Molassembler: Molecular graph construction, modification, and conformer generation for inorganic and organic molecules. J. Chem. Inf. Model. 2020, 60, 3884–3900. 10.1021/acs.jcim.0c00503. 32610018 PMC12344703

[ref23] Chernyshov I. Y.; Pidko E. A. MACE: Automated assessment of stereochemistry of transition metal complexes and its applications in computational catalysis. J. Chem. Theory Comput. 2024, 20, 2313–2320. 10.1021/acs.jctc.3c01313. 38365199 PMC10938507

[ref24] Ioannidis E. I.; Gani T. Z. H.; Kulik H. J. MolSimplify: A Toolkit for Automating Discovery in Inorganic Chemistry. J. Comput. Chem. 2016, 37, 2106–2117. 10.1002/jcc.24437. 27364957

[ref25] Foscato M.; Venkatraman V.; Jensen V. R. DENOPTIM: Software for Computational *de Novo* Design of Organic and Inorganic Molecules. J. Chem. Inf. Model. 2019, 59, 4077–4082. 10.1021/acs.jcim.9b00516. 31479254

[ref26] Jin H.; Merz K. M. LigandDiff: de Novo Ligand Design for 3D Transition Metal Complexes with Diffusion Models. J. Chem. Theory Comput. 2024, 20, 4377–4384. 10.1021/acs.jctc.4c00232. 38743854 PMC11137811

[ref27] Ho J.; Jain A.; Abbeel P. Denoising diffusion probabilistic models. In NIPS’20: Proceedings of the 34th International Conference on Neural Information Processing System; ACM, 2020; Vol. 33; pp 6840–6851.

[ref28] Hoogeboom E.; Satorras V. G.; Vignac C.; Welling M. Equivariant diffusion for molecule generation in 3d. In Proceedings of the 39th International Conference on Machine learning; PMLR, 2022, pp 8867–8887.

[ref29] Igashov I.; Stärk H.; Vignac C.; Schneuing A.; Satorras V. G.; Frossard P.; Welling M.; Bronstein M.; Correia B. Equivariant 3D-conditional diffusion model for molecular linker design. Nat. Mach. Intell. 2024, 6, 417–427. 10.1038/s42256-024-00815-9.

[ref30] Abramson J.; Adler J.; Dunger J.; Evans R.; Green T.; Pritzel A.; Ronneberger O.; Willmore L.; Ballard A. J.; Bambrick J.; Bodenstein S. W.; Evans D. A.; Hung C.-C.; O’Neill M.; Reiman D.; Tunyasuvunakool K.; Wu Z.; Žemgulytė A.; Arvaniti E.; Beattie C.; Bertolli O.; Bridgland A.; Cherepanov A.; Congreve M.; Cowen-Rivers A. I.; Cowie A.; Figurnov M.; Fuchs F. B.; Gladman H.; Jain R.; Khan Y. A.; Low C. M. R.; Perlin K.; Potapenko A.; Savy P.; Singh S.; Stecula A.; Thillaisundaram A.; Tong C.; Yakneen S.; Zhong E. D.; Zielinski M.; Žídek A.; Bapst V.; Kohli P.; Jaderberg M.; Hassabis D.; Jumper J. M. Accurate structure prediction of biomolecular interactions with AlphaFold 3. Nature 2024, 630, 493–500. 10.1038/s41586-024-07487-w. 38718835 PMC11168924

[ref31] Borissova A. O.; Antipin M. Yu.; Lyssenko K. A. Mutual Influence of Cyclopentadienyl and Carbonyl Ligands in Cymantrene: QTAIM Study. J. Phys. Chem. A 2009, 113, 10845–10851. 10.1021/jp905841r. 19754097

[ref32] Sajith P. K.; Suresh C. H. Quantification of Mutual Trans Influence of Ligands in Pd (II) Complexes: A Combined Approach Using Isodesmic Reactions and Aim Analysis. Dalton Trans. 2010, 39, 815–822. 10.1039/B911013E. 20066226

[ref33] Clough T. J.; Jiang L.; Wong K.-L.; Long N. J. Ligand Design Strategies to Increase Stability of Gadolinium-Based Magnetic Resonance Imaging Contrast Agents. Nat. Commun. 2019, 10, 1420 10.1038/s41467-019-09342-3. 30926784 PMC6441101

[ref34] Saeedifard F.; Breyer C. J.; Chi Cao T.; Kamdar J. M.; Kerkhof J.; Smith D. K.; Cooksy A. L.; Rheingold A. L.; Moore C. E.; Gu J.; Grotjahn D. B. Increasing Ligand Denticity and Stability for a Water Oxidation Electrocatalyst Using P(V) as Connecting Element. ChemCatChem 2024, 16, e202301644 10.1002/cctc.202301644.

[ref35] Toporivska Y.; Mular A.; Piasta K.; Ostrowska M.; Illuminati D.; Baldi A.; Albanese V.; Pacifico S.; Fritsky I. O.; Remelli M.; Guerrini R.; Gumienna-Kontecka E. Thermodynamic Stability and Speciation of Ga(III) and Zr(Iv) Complexes with High Denticity Hydroxamate Chelators. Inorg. Chem. 2021, 60, 13332–13347. 10.1021/acs.inorgchem.1c01622. 34414758 PMC8424644

[ref36] Smits N. W.; van Dijk B.; de Bruin I.; Groeneveld S. L.; Siegler M. A.; Hetterscheid D. G. Influence of Ligand Denticity and Flexibility on the Molecular Copper Mediated Oxygen Reduction Reaction. Inorg. Chem. 2020, 59, 16398–16409. 10.1021/acs.inorgchem.0c02204. 33108871 PMC7672700

[ref37] Deka H.; Ghosh S.; Saha S.; Gogoi K.; Mondal B. Effect of Ligand Denticity on the Nitric Oxide Reactivity of Cobalt(II) Complexes. Dalton Trans. 2016, 45, 10979–10988. 10.1039/C6DT01169A. 27305969

[ref38] Preston D.; Kruger P. E. Using Complementary Ligand Denticity to Direct Metallosupramolecular Structure about Metal Ions with Square-planar Geometry. ChemPlusChem 2020, 85, 454–465. 10.1002/cplu.202000019. 32159301

[ref39] Meagley K. L.; Garcia S. P. Chemical Control of Crystal Growth with Multidentate Carboxylate Ligands: Effect of Ligand Denticity on Zinc Oxide Crystal Shape. Cryst. Growth Des. 2012, 12, 707–713. 10.1021/cg200992z.

[ref40] Paprocka R.; Wiese-Szadkowska M.; Janciauskiene S.; Kosmalski T.; Kulik M.; Helmin-Basa A. Latest Developments in Metal Complexes as Anticancer Agents. Coord. Chem. Rev. 2022, 452, 214307 10.1016/j.ccr.2021.214307.

[ref41] Needham R. J.; Bridgewater H. E.; Romero-Canelón I.; Habtemariam A.; Clarkson G. J.; Sadler P. J. Structure-Activity Relationships for Osmium(II) Arene Phenylazopyridine Anticancer Complexes Functionalised with Alkoxy and Glycolic Substituents. J. Inorg. Biochem. 2020, 210, 111154 10.1016/j.jinorgbio.2020.111154. 32771772

[ref42] Fu Y.; Habtemariam A.; Basri A. M. B. H.; Braddick D.; Clarkson G. J.; Sadler P. J. Structure–Activity Relationships for Organometallic Osmium Arene Phenylazopyridine Complexes with Potent Anticancer Activity. Dalton Trans. 2011, 40, 10553 10.1039/c1dt10937e. 21860862

[ref43] Valdés H.; García-Eleno M. A.; Canseco-Gonzalez D.; Morales-Morales D. Recent Advances in Catalysis with Transition-metal Pincer Compounds. ChemCatChem 2018, 10, 3136–3172. 10.1002/cctc.201702019.

[ref44] Saha S.; Assanar F.; Ghosh S. Transition Metal Borate Complexes Containing κ^0^-κ^6^ Denticity of Scorpionate Borate Ligands. Eur. J. Inorg. Chem. 2022, 26, e202200587 10.1002/ejic.202200587.

[ref45] Halcrow M. A. Spin-Crossover Materials: Properties and Applications; John Wiley& Sons, Ltd, 2013.

[ref46] Cirera J.; Via-Nadal M.; Ruiz E. Benchmarking Density Functional Methods for Calculation of State Energies of First Row Spin-Crossover Molecules. Inorg. Chem. 2018, 57, 14097–14105. 10.1021/acs.inorgchem.8b01821. 30383364

[ref47] Senthil Kumar K.; Ruben M. Emerging Trends in Spin Crossover (SCO) Based Functional Materials and Devices. Coord. Chem. Rev. 2017, 346, 176–205. 10.1016/j.ccr.2017.03.024.

[ref48] Kumar K. S.; Ruben M. Sublimable Spin-crossover Complexes: From Spin-state Switching to Molecular Devices. Angew. Chem., Int. Ed. 2021, 60, 7502–7521. 10.1002/anie.201911256. PMC8048919 31769131

[ref49] Nandy A.; Taylor M. G.; Kulik H. J. Identifying underexplored and untapped regions in the chemical space of transition metal complexes. J. Phys. Chem. Lett. 2023, 14, 5798–5804. 10.1021/acs.jpclett.3c01214. 37338110

[ref50] Arunachalam N.; Gugler S.; Taylor M. G.; Duan C.; Nandy A.; Janet J. P.; Meyer R.; Oldenstaedt J.; Chu D. B. K.; Kulik H. J. Ligand additivity relationships enable efficient exploration of transition metal chemical space. J. Chem. Phys. 2022, 157, 184112 10.1063/5.0125700. 36379790

[ref51] Jing B., Eismann S., Suriana P., Townshend R. J. L., Dror R. Learning from Protein Structure with Geometric Vector Perceptrons. 2021, arXiv: 2009.01411. arXiv preprint. https://doi.org/10.48550/arXiv.2009.01411 (accessed April 24, 2024).

[ref52] Jing B., Eismann S., Soni P. N., Dror R. O. Equivariant Graph Neural Networks for 3D Macromolecular Structure. 2021, arXiv: 2106.03843. arXiv preprint. https://arxiv.org/abs/2106.03843 (accessed April 24, 2024).

[ref53] Torge J., Harris C., Mathis S. V., Lio P. Diffhopp: A graph diffusion model for novel drug design via scaffold hopping. 2023, arXiv:2308.07416. https://doi.org/10.48550/arXiv.2308.07416 (accessed April 24, 2024).

[ref54] Vennelakanti V.; Taylor M. G.; Nandy A.; Duan C.; Kulik H. J. Assessing the Performance of Approximate Density Functional Theory on 95 Experimentally Characterized Fe (II) Spin Crossover Complexes. J. Chem. Phys. 2023, 159, 024120 10.1063/5.0157187. 37431914

[ref55] Bannwarth C.; Caldeweyher E.; Ehlert S.; Hansen A.; Pracht P.; Seibert J.; Spicher S.; Grimme S. Extended Tight-binding Quantum Chemistry Methods. Wiley Interdiscip. Rev.: Comput. Mol. Sci. 2021, 11, e1493 10.1002/wcms.1493.

[ref56] Brandenburg J. G.; Bannwarth C.; Hansen A.; Grimme S. B97-3C: A Revised Low-Cost Variant of the B97-D Density Functional Method. J. Chem. Phys. 2018, 148, 064104 10.1063/1.5012601. 29448802

[ref57] Neese F. Software Update: The Orca Program System—Version 5.0. Wiley Interdiscip. Rev.: Comput. Mol. Sci. 2022, 12, e1606 10.1002/wcms.1606.

[ref58] Jin H.; Merz K. M. Modeling Fe (II) Complexes Using Neural Networks. J. Chem. Theory Comput. 2024, 20, 2551–2558. 10.1021/acs.jctc.4c00063. 38439716 PMC10976644

[ref59] Jin H.; Merz K. M. Modeling Zinc Complexes Using Neural Networks. J. Chem. Inf. Model. 2024, 64, 3140–3148. 10.1021/acs.jcim.4c00095. 38587510 PMC11040731

[ref60] Staroverov V. N.; Scuseria G. E.; Tao J.; Perdew J. P. Comparative Assessment of a New Nonempirical Density Functional: Molecules and Hydrogen-Bonded Complexes. J. Chem. Phys. 2003, 119, 12129–12137. 10.1063/1.1626543.

[ref61] Caldeweyher E.; Ehlert S.; Hansen A.; Neugebauer H.; Spicher S.; Bannwarth C.; Grimme S. A Generally Applicable Atomic-Charge Dependent London Dispersion Correction. J. Chem. Phys. 2019, 150, 154122 10.1063/1.5090222. 31005066

[ref62] Weigend F.; Ahlrichs R. Balanced Basis Sets of Split Valence, Triple Zeta Valence and Quadruple Zeta Valence Quality for H to RN: Design and Assessment of Accuracy. Phys. Chem. Chem. Phys. 2005, 7, 3297–3305. 10.1039/b508541a. 16240044

[ref63] Neese F. An Improvement of the Resolution of the Identity Approximation for the Formation of the Coulomb Matrix. J. Comput. Chem. 2003, 24, 1740–1747. 10.1002/jcc.10318. 12964192

[ref64] Weigend F. Accurate Coulomb-Fitting Basis Sets for H to Rn. Phys. Chem. Chem. Phys. 2006, 8 (9), 1057–1065. 10.1039/b515623h. 16633586

