# Supplementary material for: Partial to Total Generation of 3D Transition-Metal Complexes
Source: J Chem Theory Comput. 2024 Sep 9;20(18):8367–77. doi: 10.1021/acs.jctc.4c00775 (PMC11428130; doi:10.1021/acs.jctc.4c00775)
Supplement: Supplementary file 1 — ct4c00775_si_001.pdf [file ct4c00775_si_001.pdf]

Supporting Information Partial to Total Generation of 3D Transition Metal Complexes

Hongni Jin[a] and Kenneth M. Merz, Jr.[a,b]*

[a]Department of Chemistry, Michigan State University,

East Lansing, Michigan 48824, United States

[b]Department of Biochemistry and Molecular Biology, Michigan State University,

East Lansing, Michigan 48824, United States

*Email: merz@chemistry.msu.edu

Table S1. The full list of LD_g

CN_c	CN_g	LD_g
0	6	111111, 11112, 1122, 1113, 114, 123, 222, 15, 24, 33, 6
1	5	11111, 1112, 113,122, 14, 23, 5
2	4	1111, 112, 22, 13, 4
3	3	111, 12, 3
4	2	11, 2
5	1	1

Table S2. The ligand denticity of 47 selected SCO complexes

Ligand denticity	Count
1,1,1,1,1,1	7
1,1,1,3	1
1,1,2,2	2
1,1,4	1
1,5	1
2,2,2	8
2,4	5
3,3	13
6	9

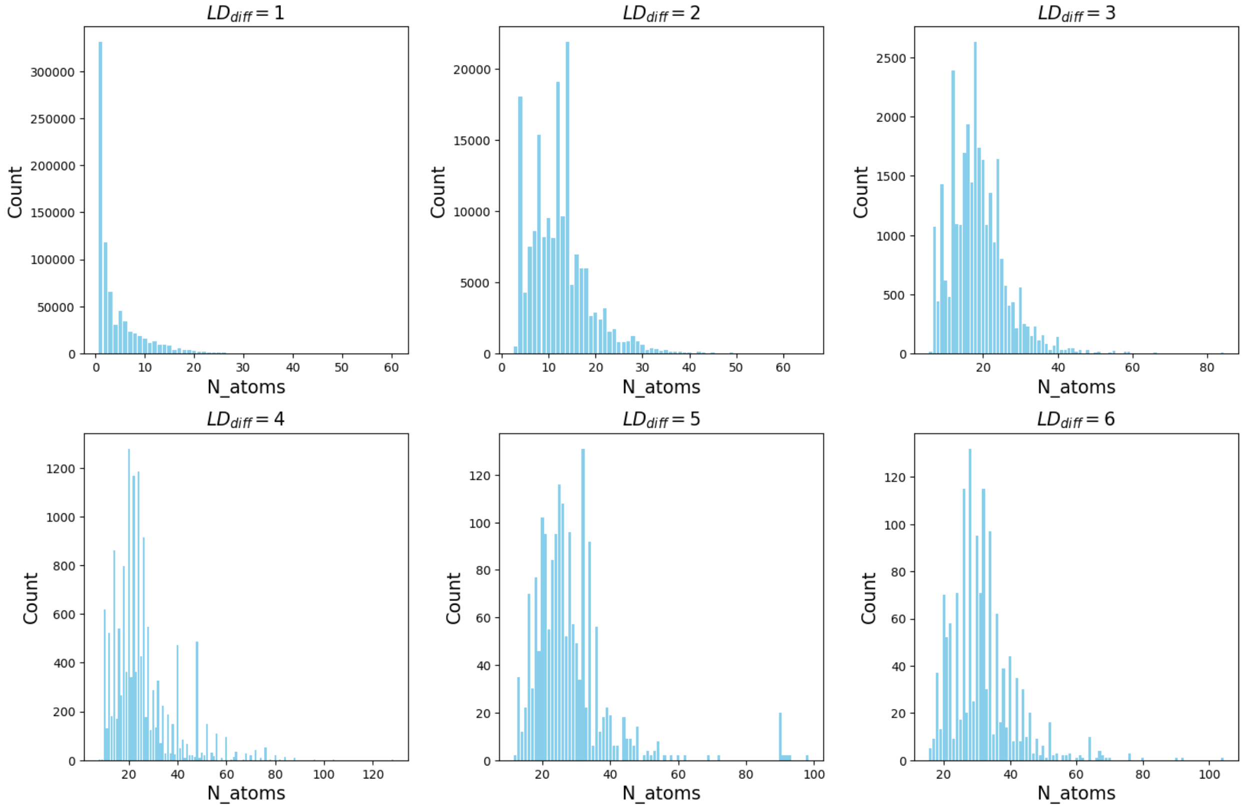

Figure S1. The size distribution of diffused ligands.

Table S3. The list of LD_g for non-octahedral geometries.

CN_c	LD_g
0	11111,1112,122,113,14,23,5<br>1111, 112, 13, 22, 4,<br>111,12,3
1	1111, 112, 13, 22, 4,<br>111,12,3,<br>11,2
2	111, 12, 3,<br>11,2,<br>1
3	11, 2,<br>1
4	1

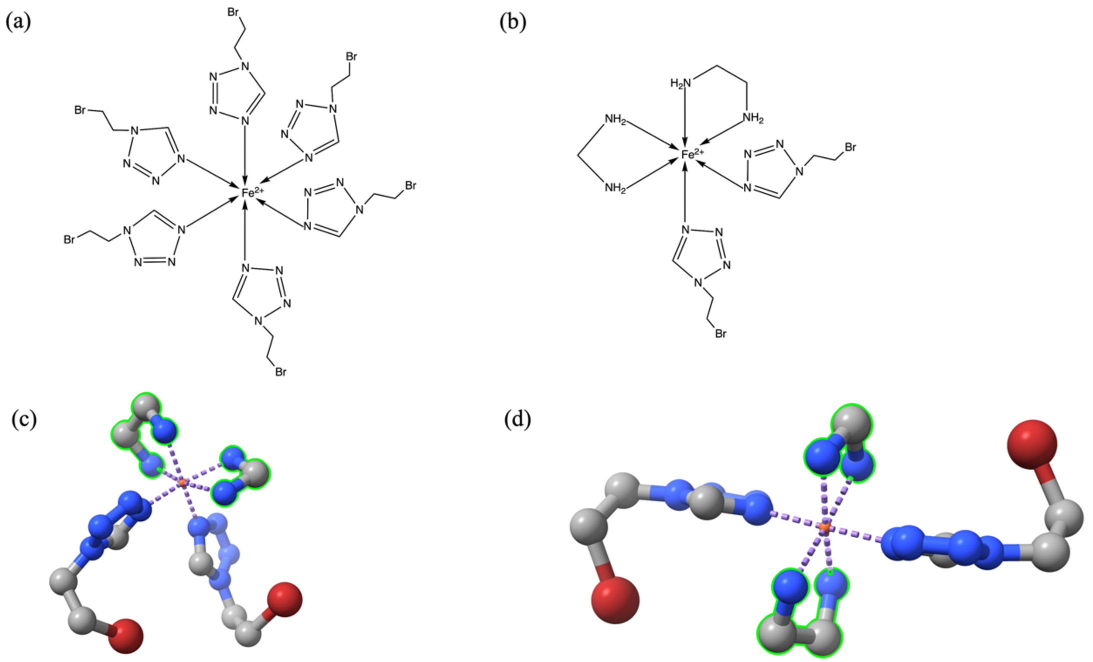

$\Delta E_{HS-LS} = 7.70 kcal/mol$ $\Delta E_{HS-LS} = 8.33 kcal/mol$

Figure S2. SCO conformers. (a)2D reference structure (refcode: YAGYIP) (b) 2D generated derivatives of reference structure. (c)(d) 3D structures of derivatives in LS state, RMSD: (c) vs. (d) = 3.62Å.

Table S4. The ligand denticity of 71 Fe(II) SCO complexes from total generation

Ligand denticity	Count
1,2,2	1
1,1,1,1,1,1	9
1,1,1,1,2	6
1,1,2,2	19
1,1,1,3	3
1,1,4	2
1,2,3	18
2,2,2	6
2,4	1
3,3	6
